# Supplementary material for: A Neoteric Feature Extraction Technique to Predict the Survival of Gastric Cancer Patients
Source: Diagnostics (Basel). 2024 May 1;14(9):954. doi: 10.3390/diagnostics14090954 (PMC11083029; doi:10.3390/diagnostics14090954)
Supplement: Supplementary file 1 [file diagnostics-14-00954-s001.zip › diagnostics-2840379-supplementary.pdf]

A Neoteric Feature Extraction Technique to Predict Survival of Gastric Cancer Patients

Warid Islam [1], Neman Abdoli [1], Tasfiq E. Alam [2], Meredith Jones [3], Bornface M. Mutembei [3], Feng Yan [3] and Qinggong Tang [3]*

[1] School of Electrical & Computer Engineering, University of Oklahoma, Norman, OK 73019, USA
[2] School of Industrial & Systems Engineering, University of Oklahoma, Norman, OK 73019, USA
[3] Stephenson School of Biomedical Engineering, University of Oklahoma, Norman, OK 73019, USA
* Correspondence: qtang@ou.edu (Q.T.)

Below are some example images of patients with gastric cancer.

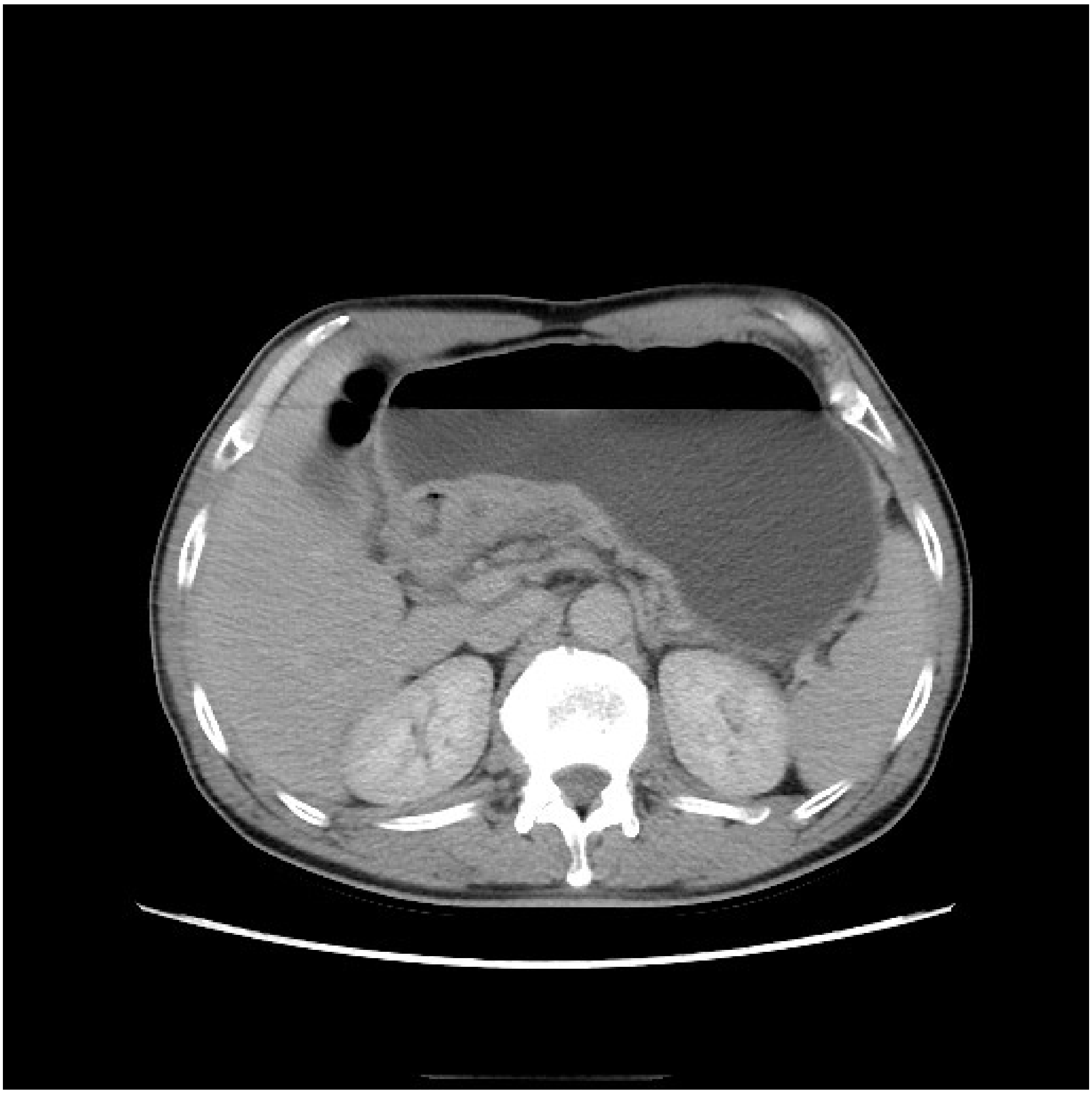

(**a**)

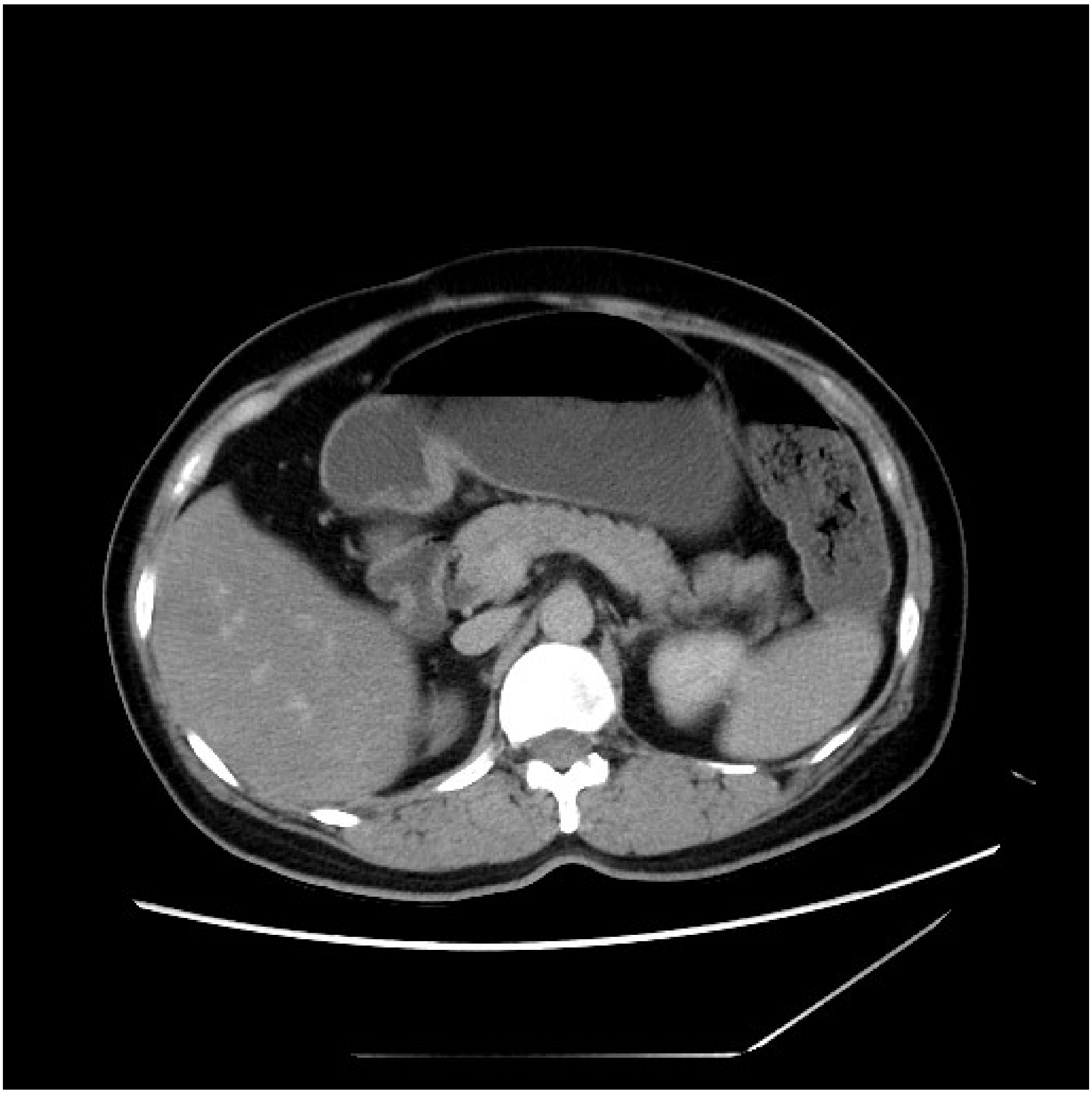

(**b**)

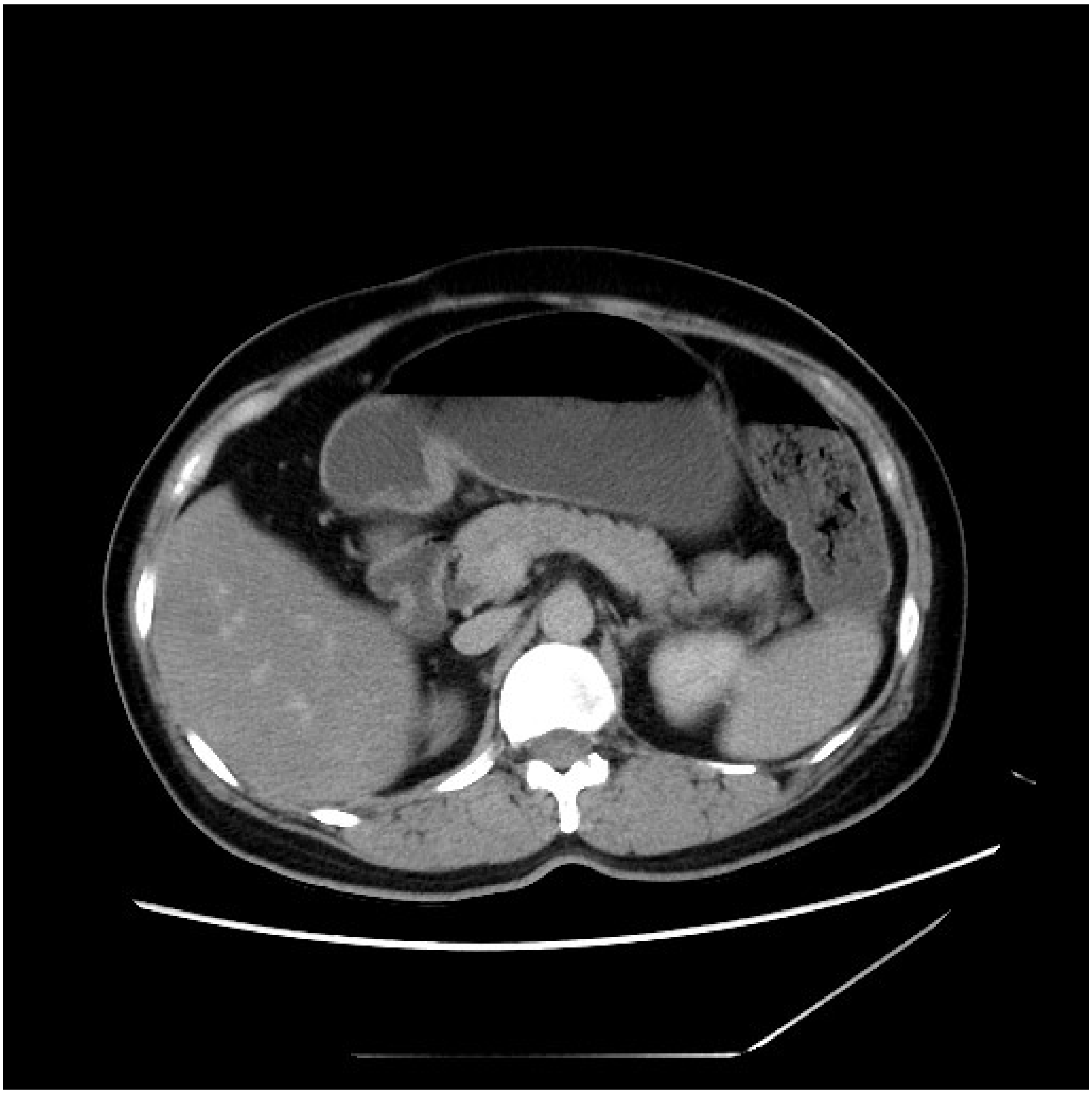

(**c**)

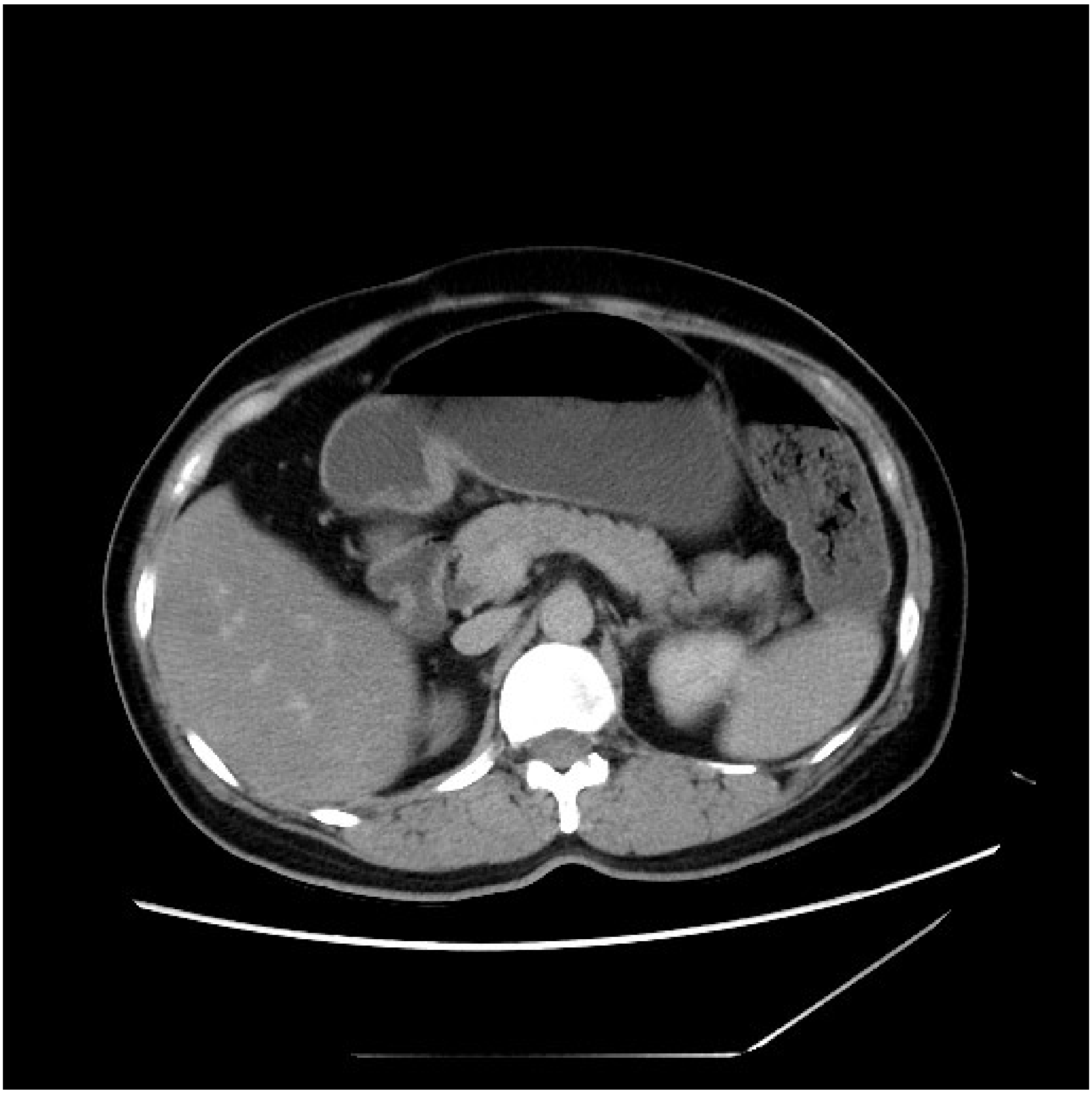

(**d**)

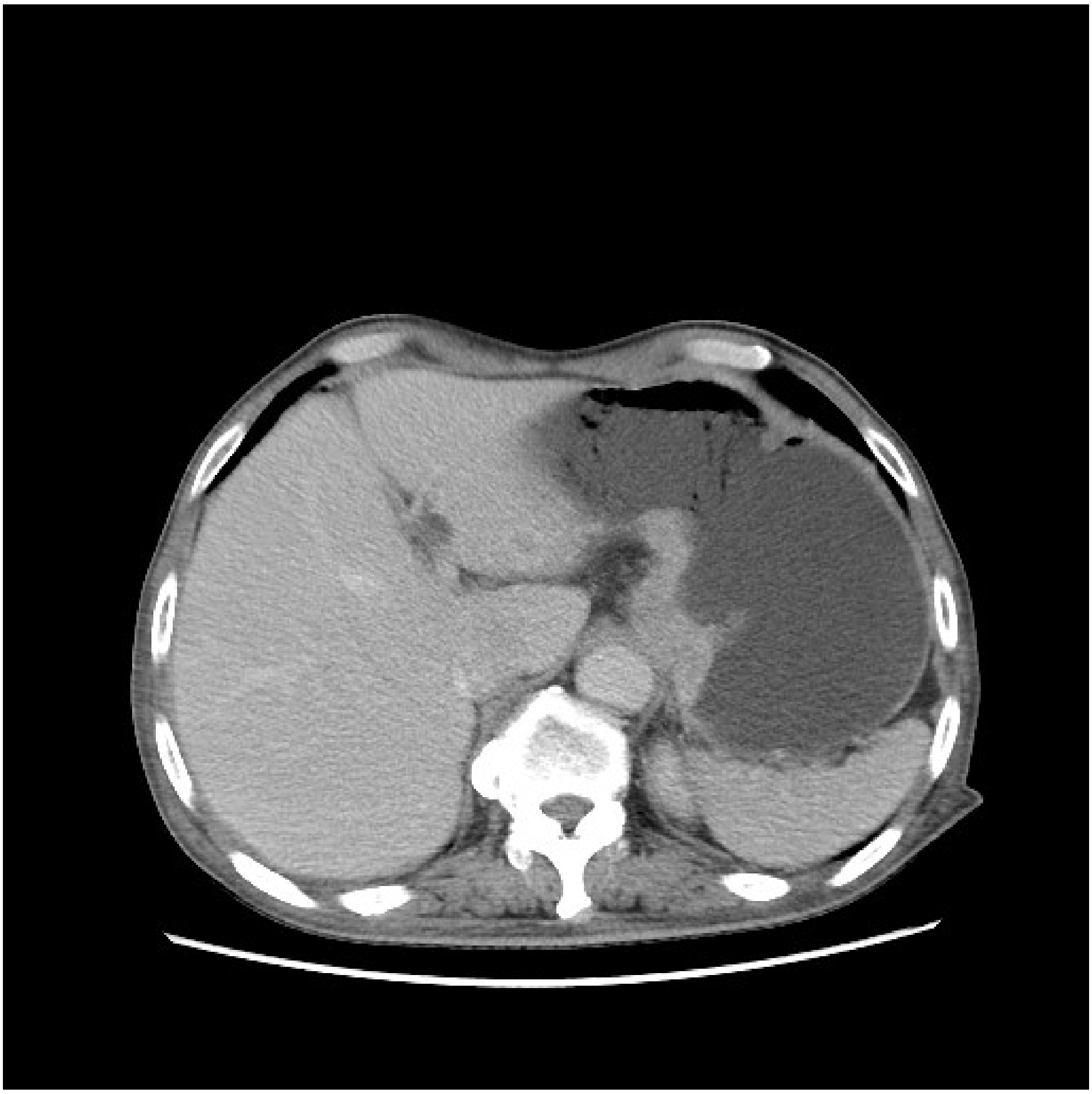

(**e**)

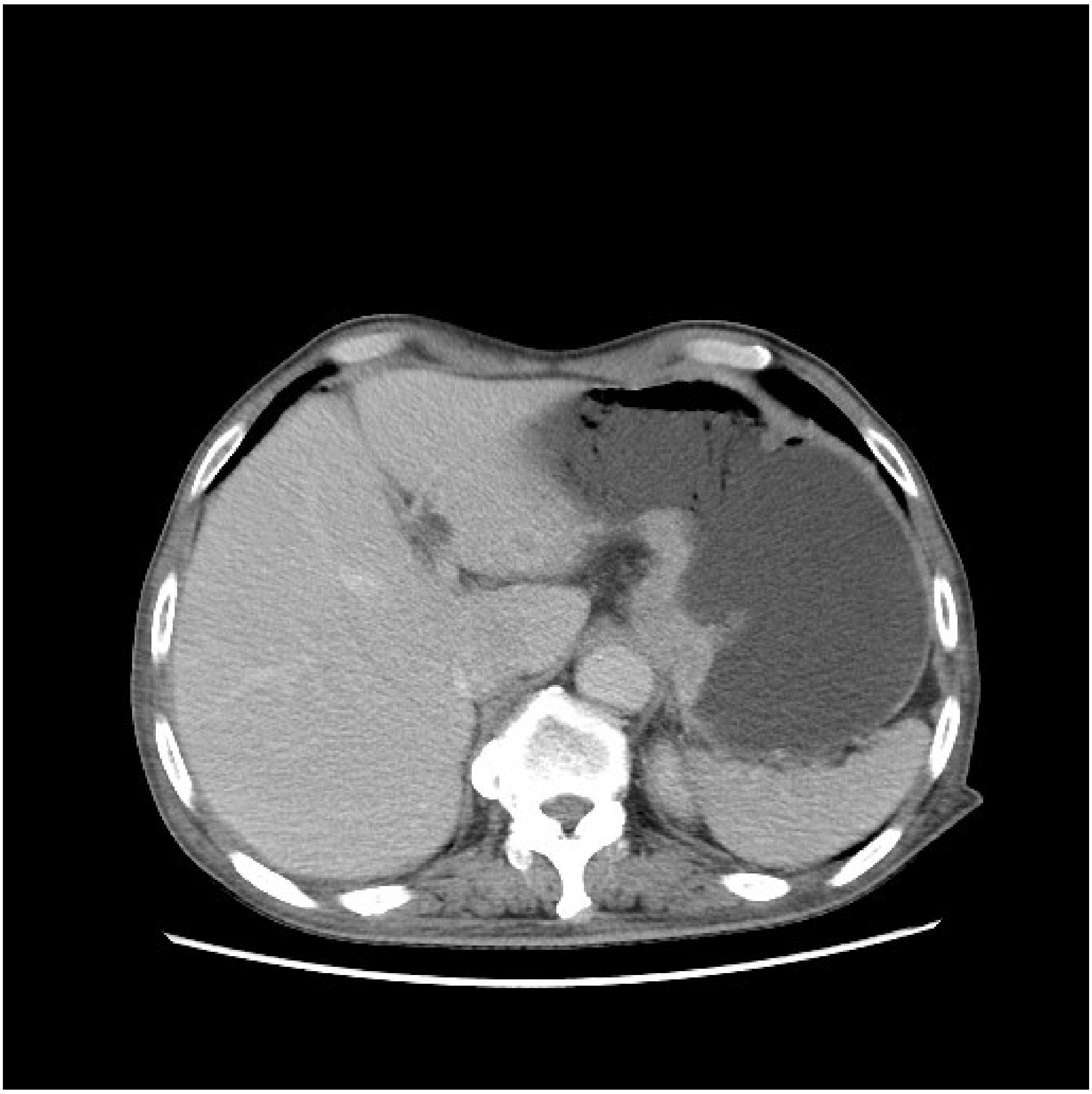

(f)

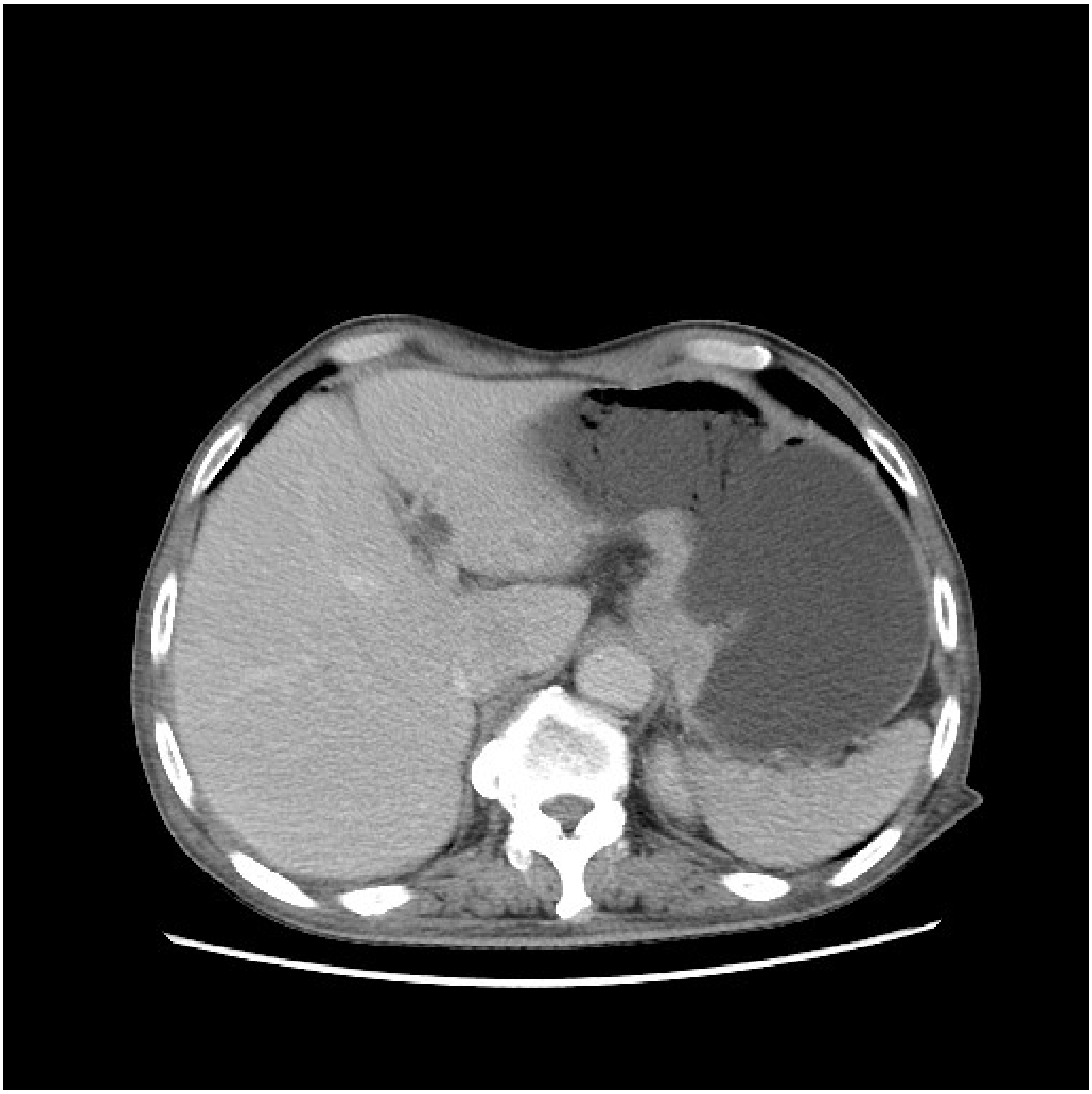

(g)

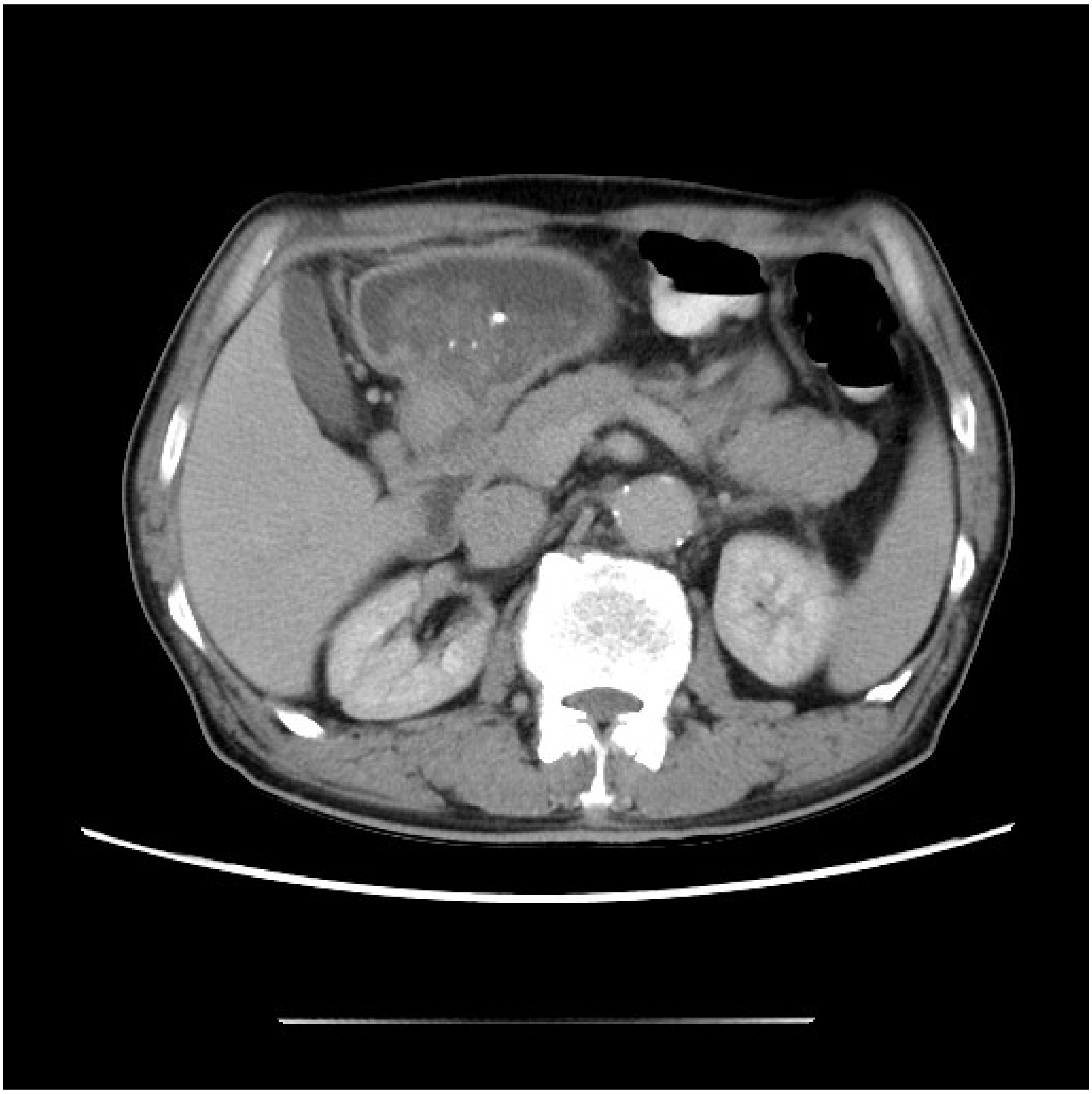

(**h**)

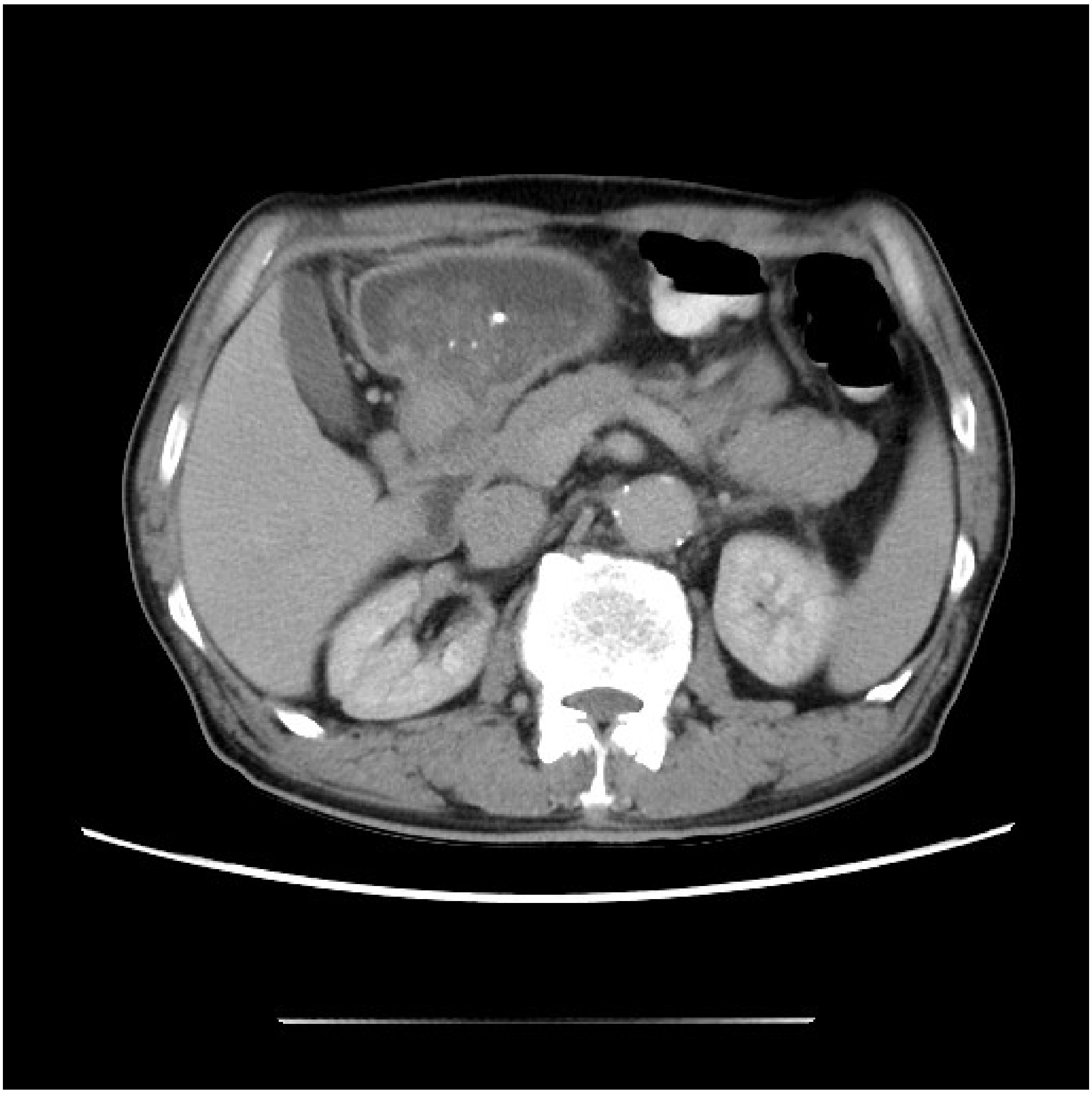

(i)

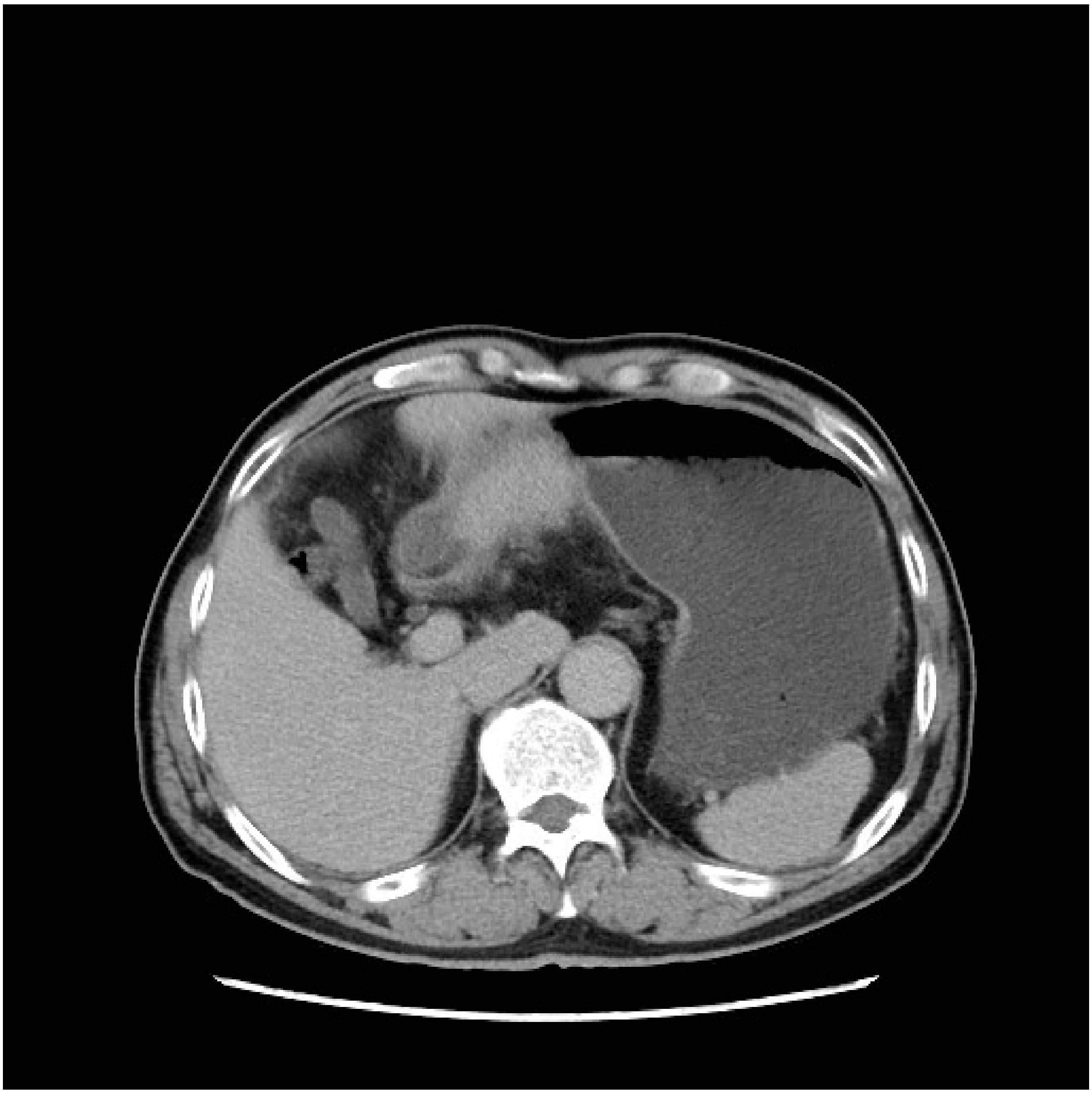

(j)

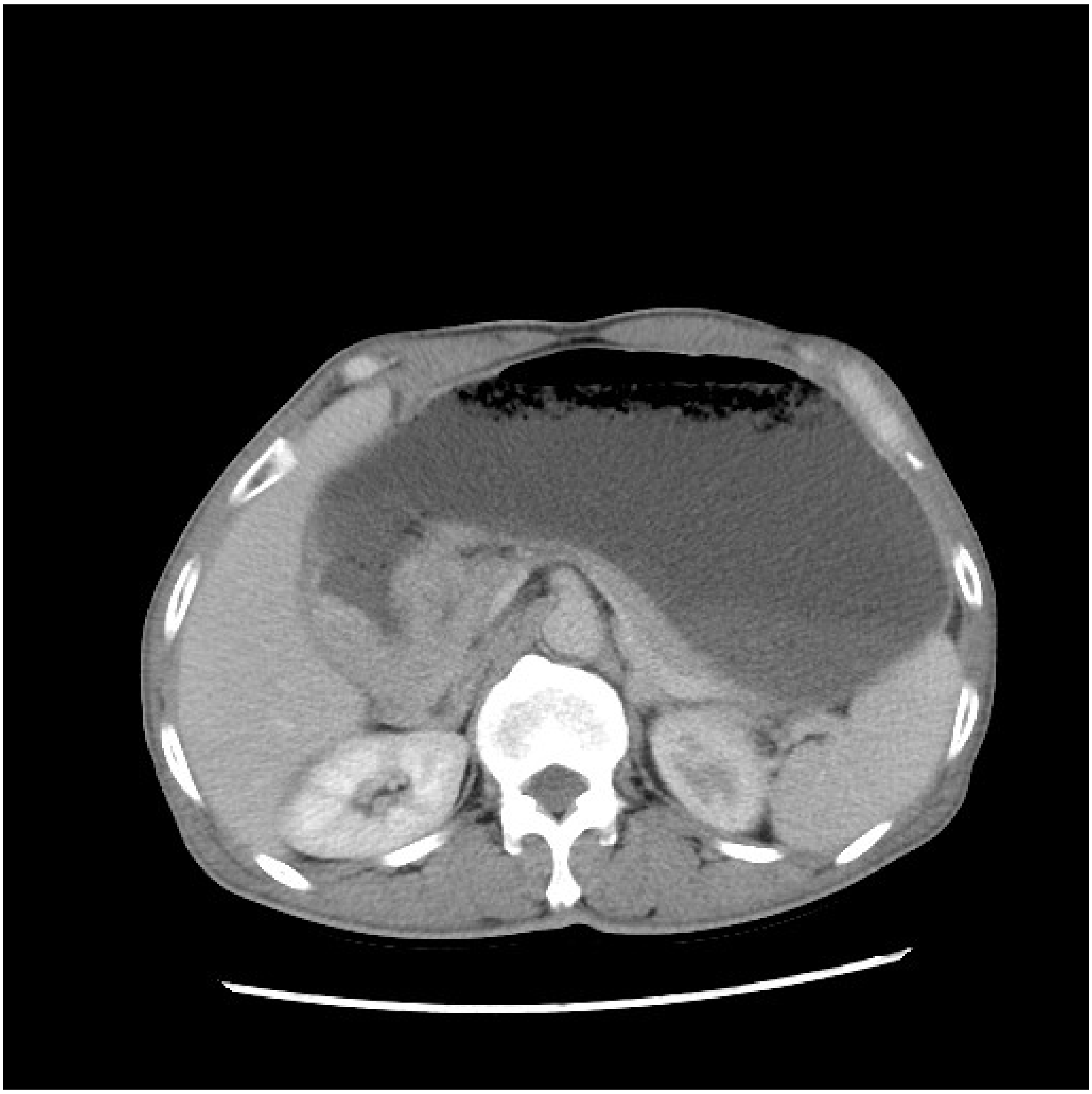

(**k**)

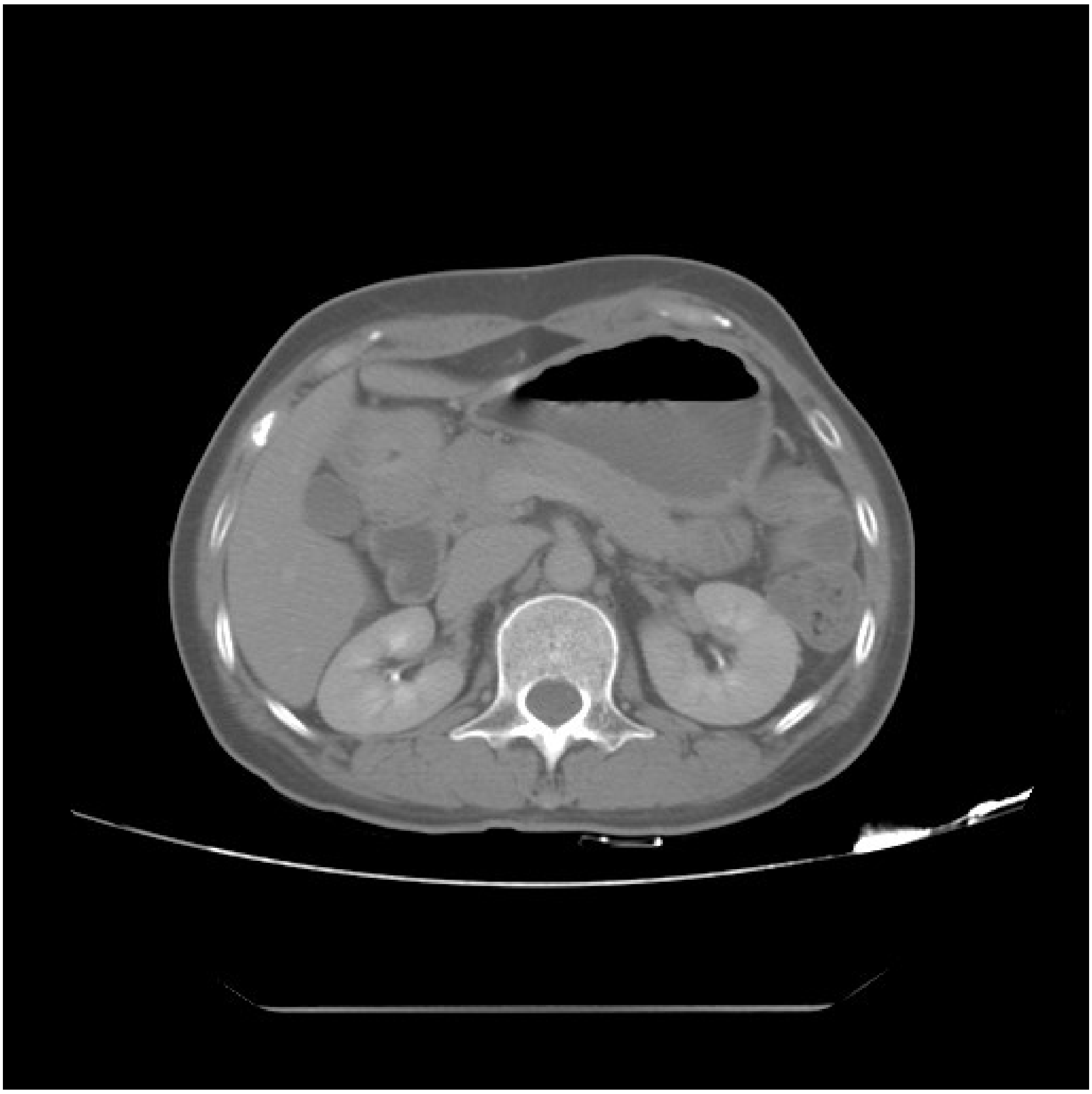

(l)

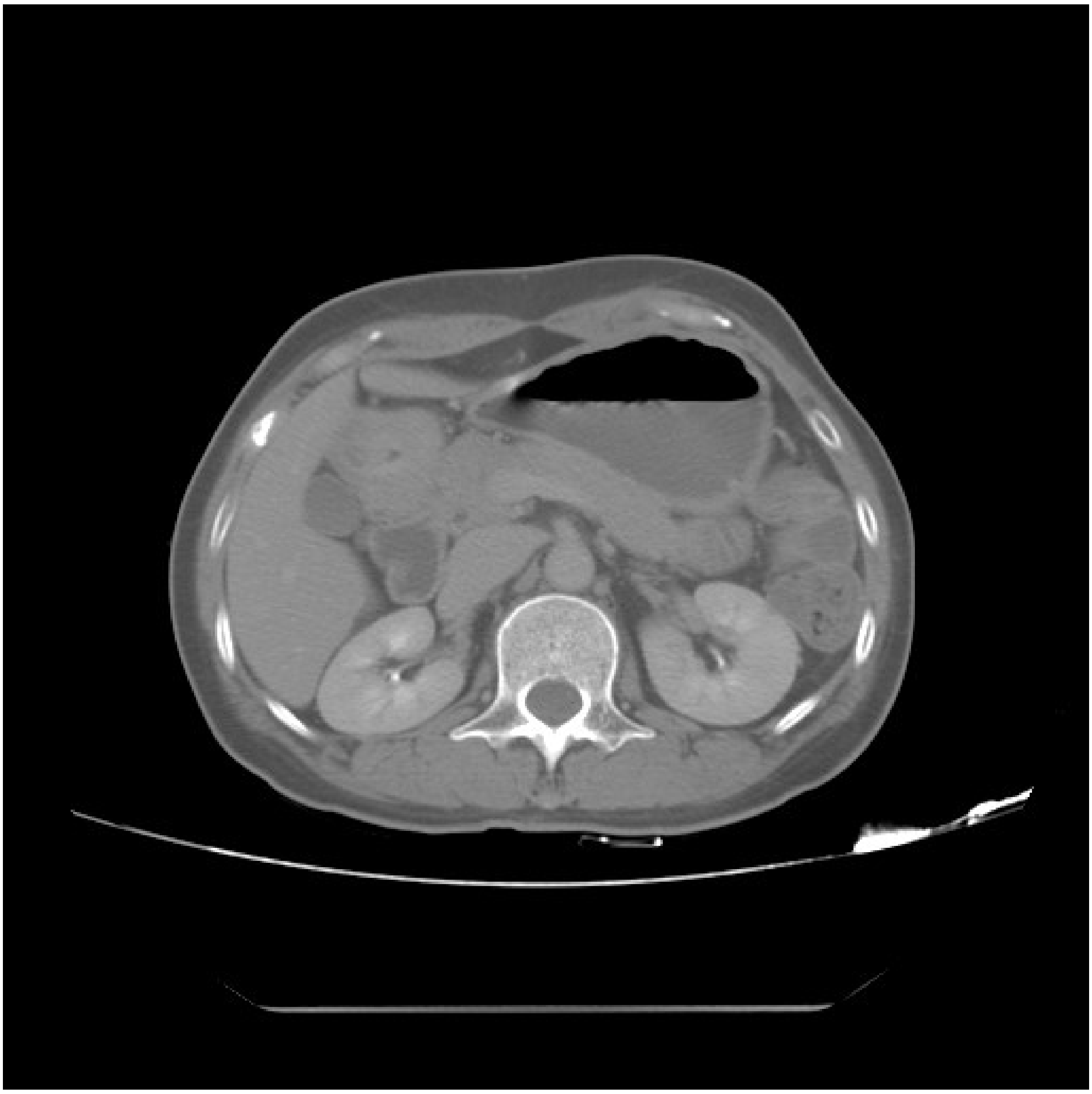

(**m**)

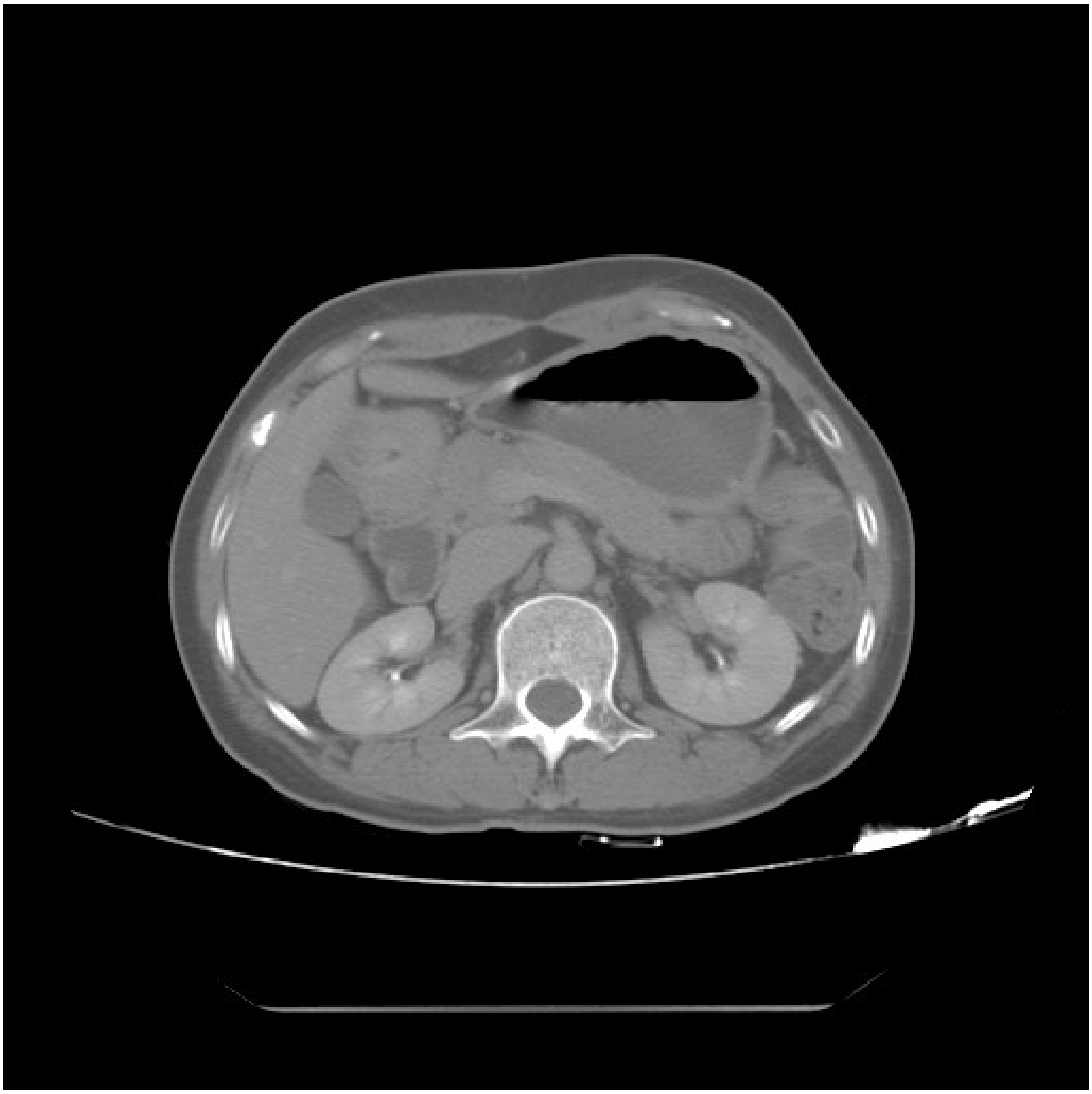

(**n**)

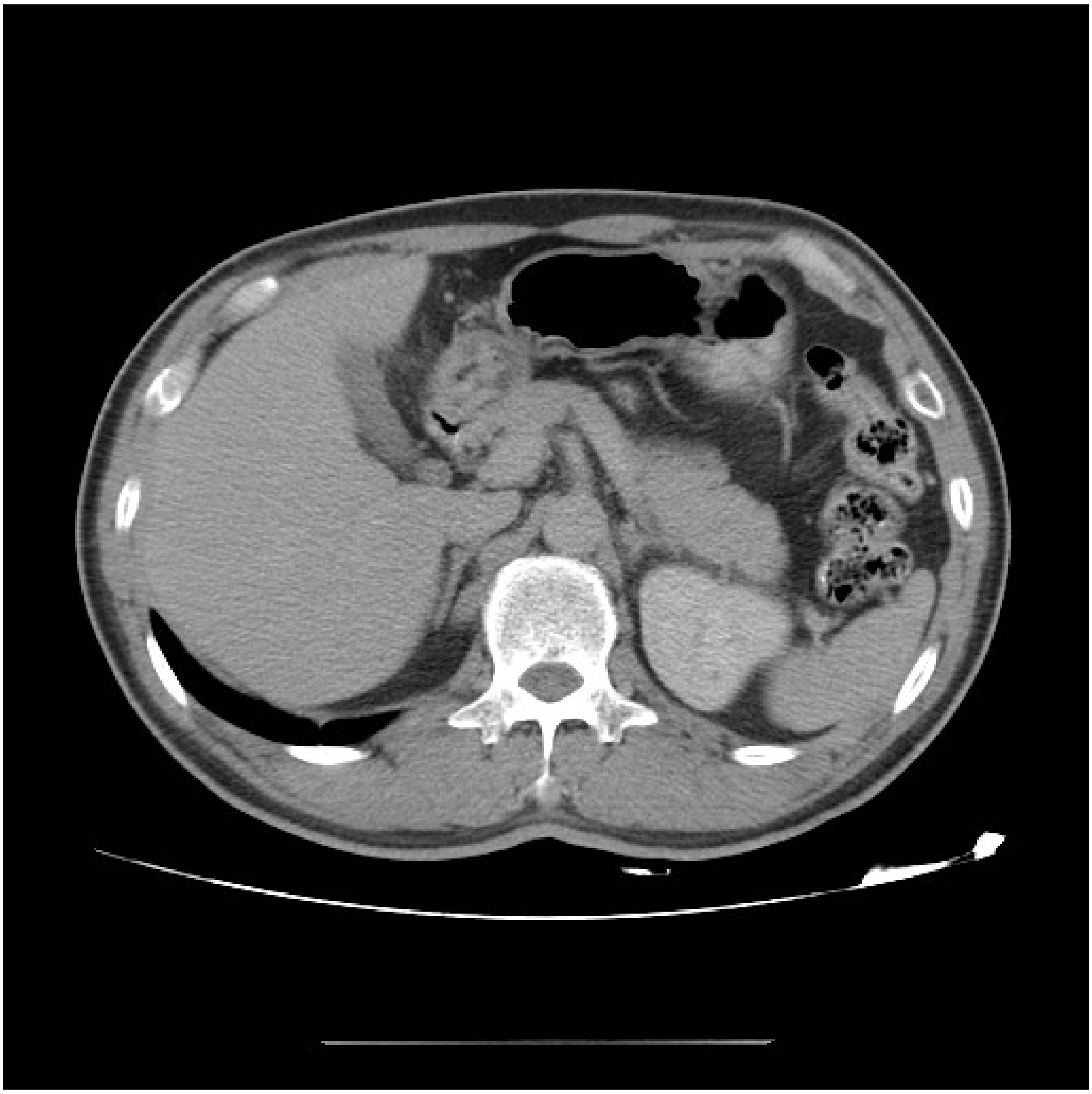

(o)

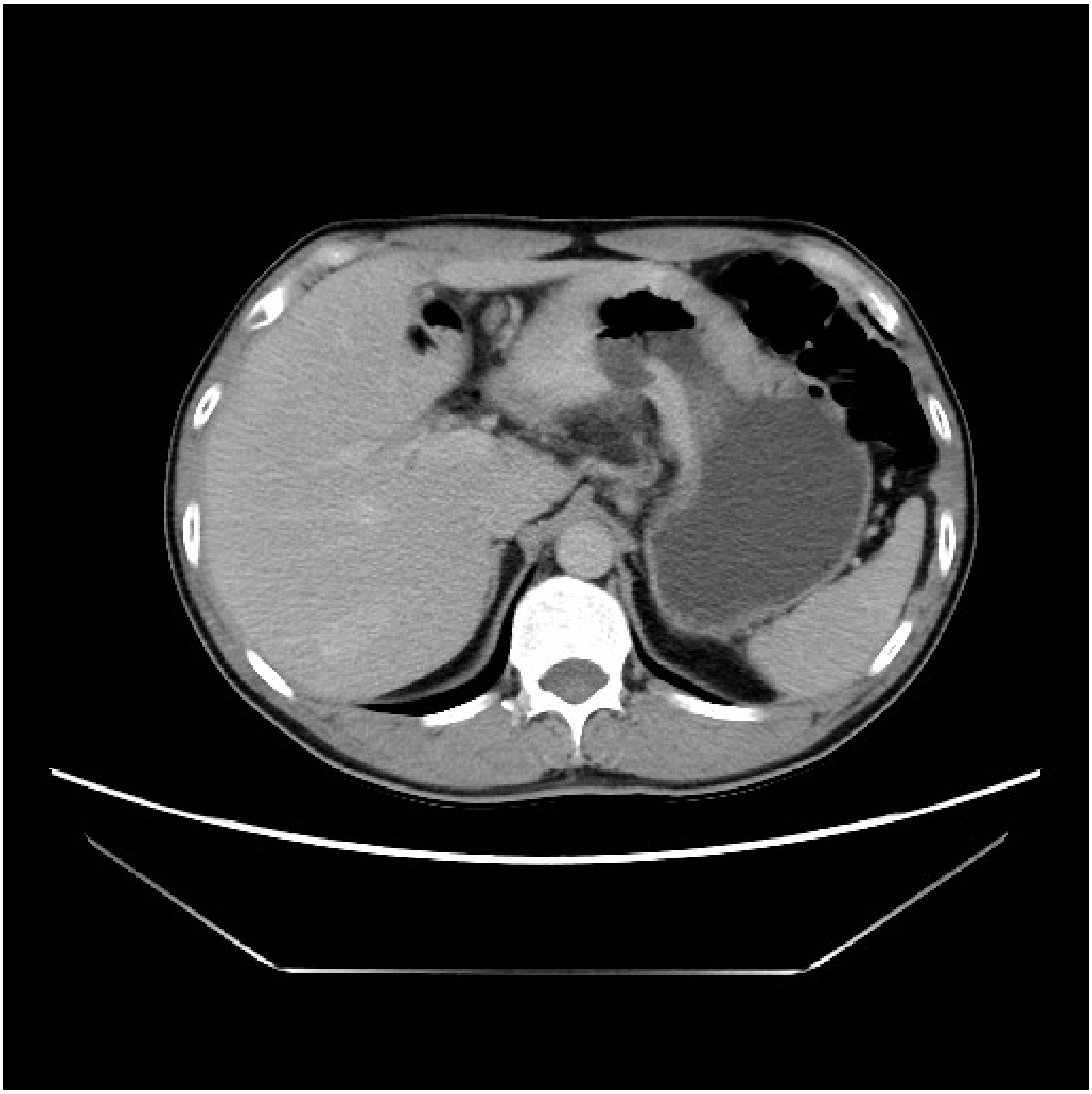

(**p**)

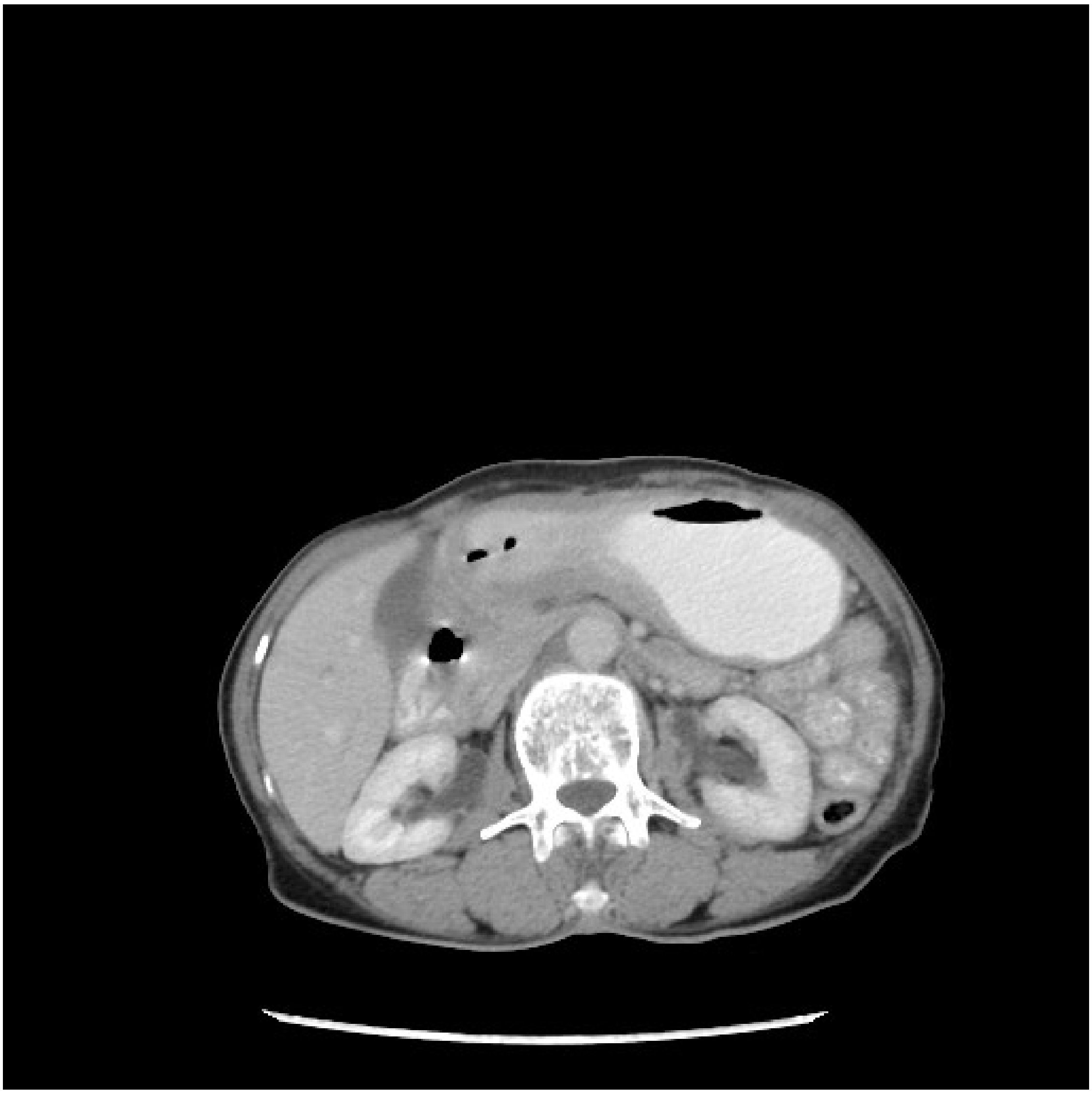

(q)

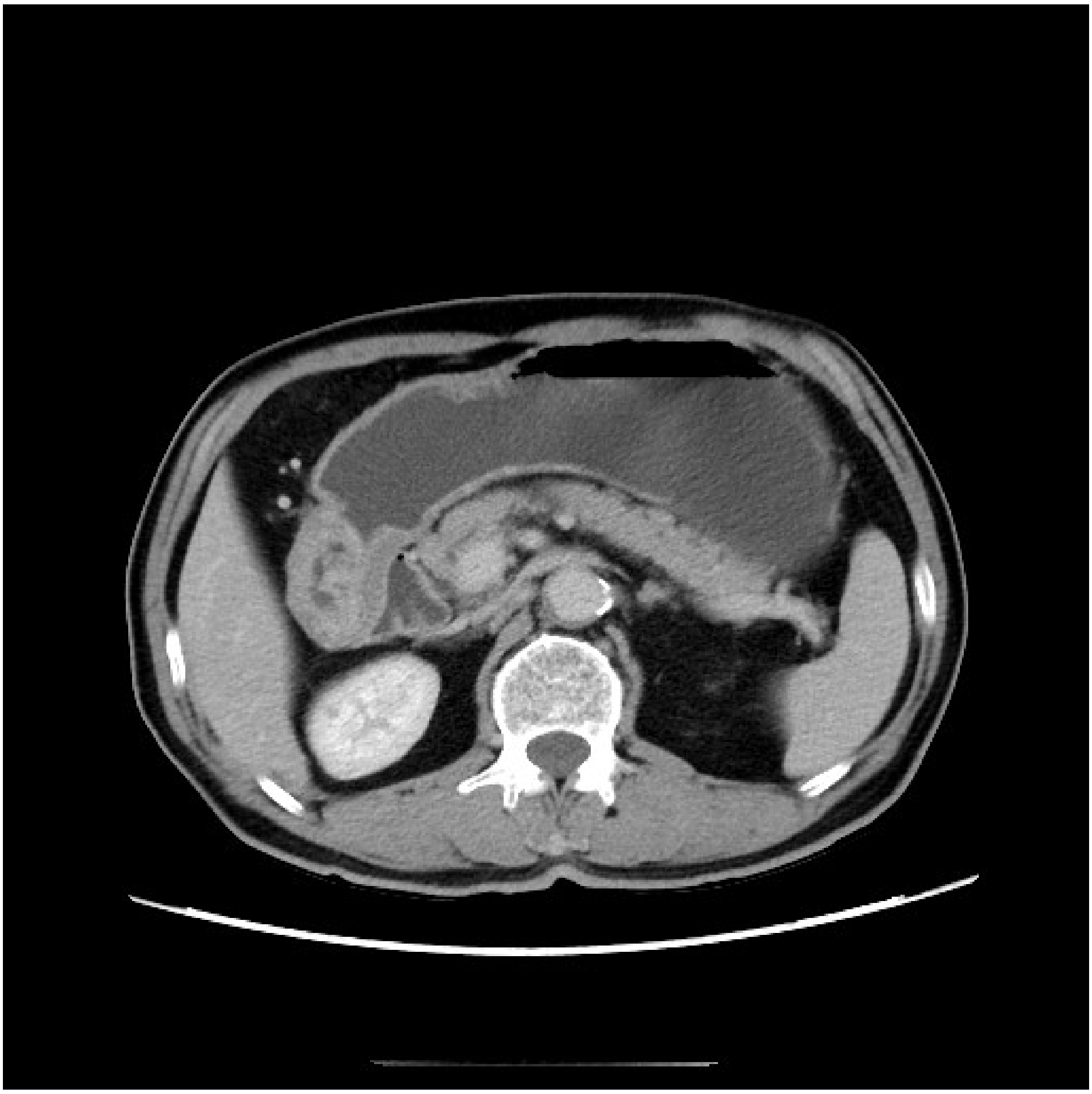

(r)

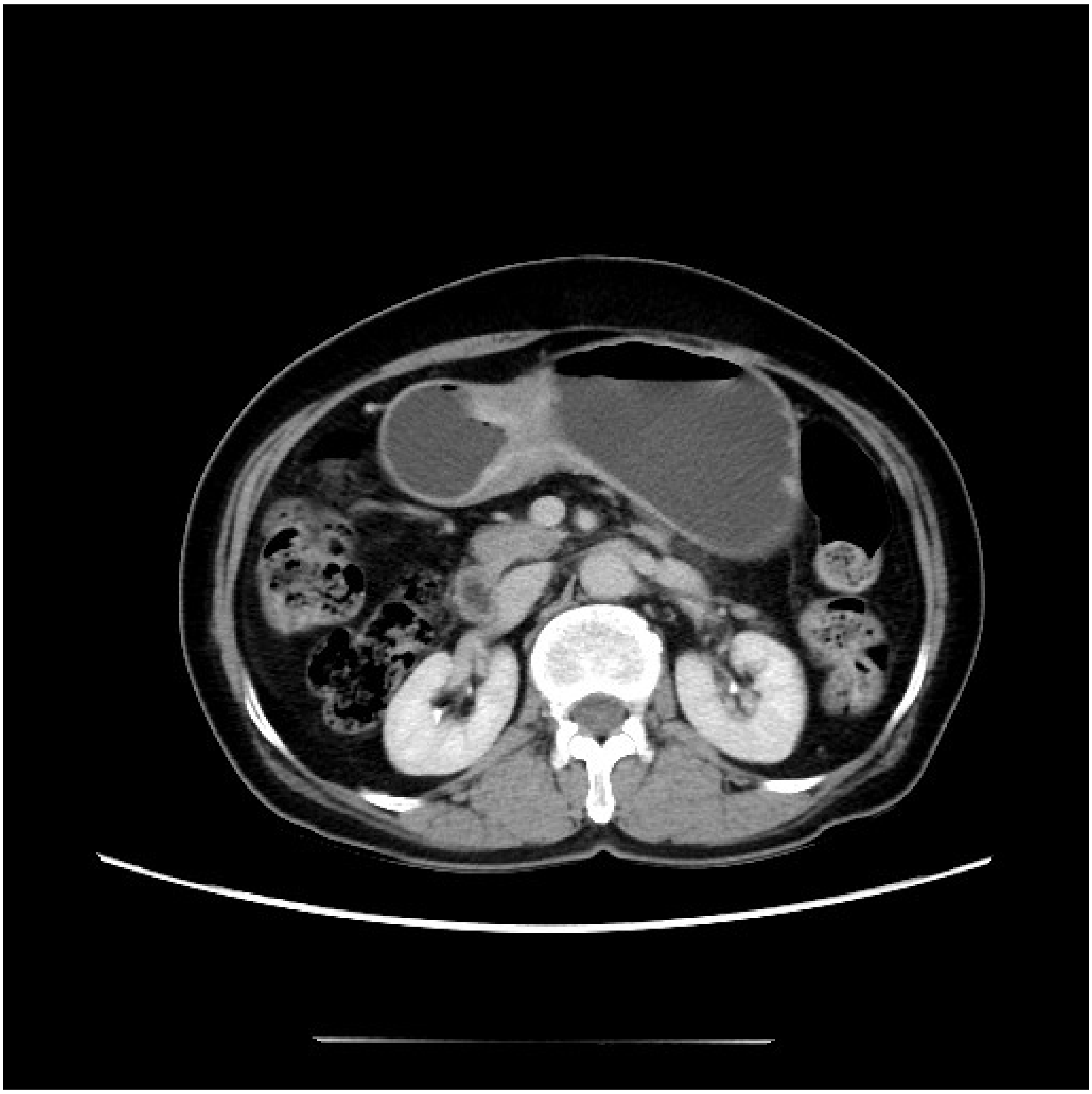

(s)

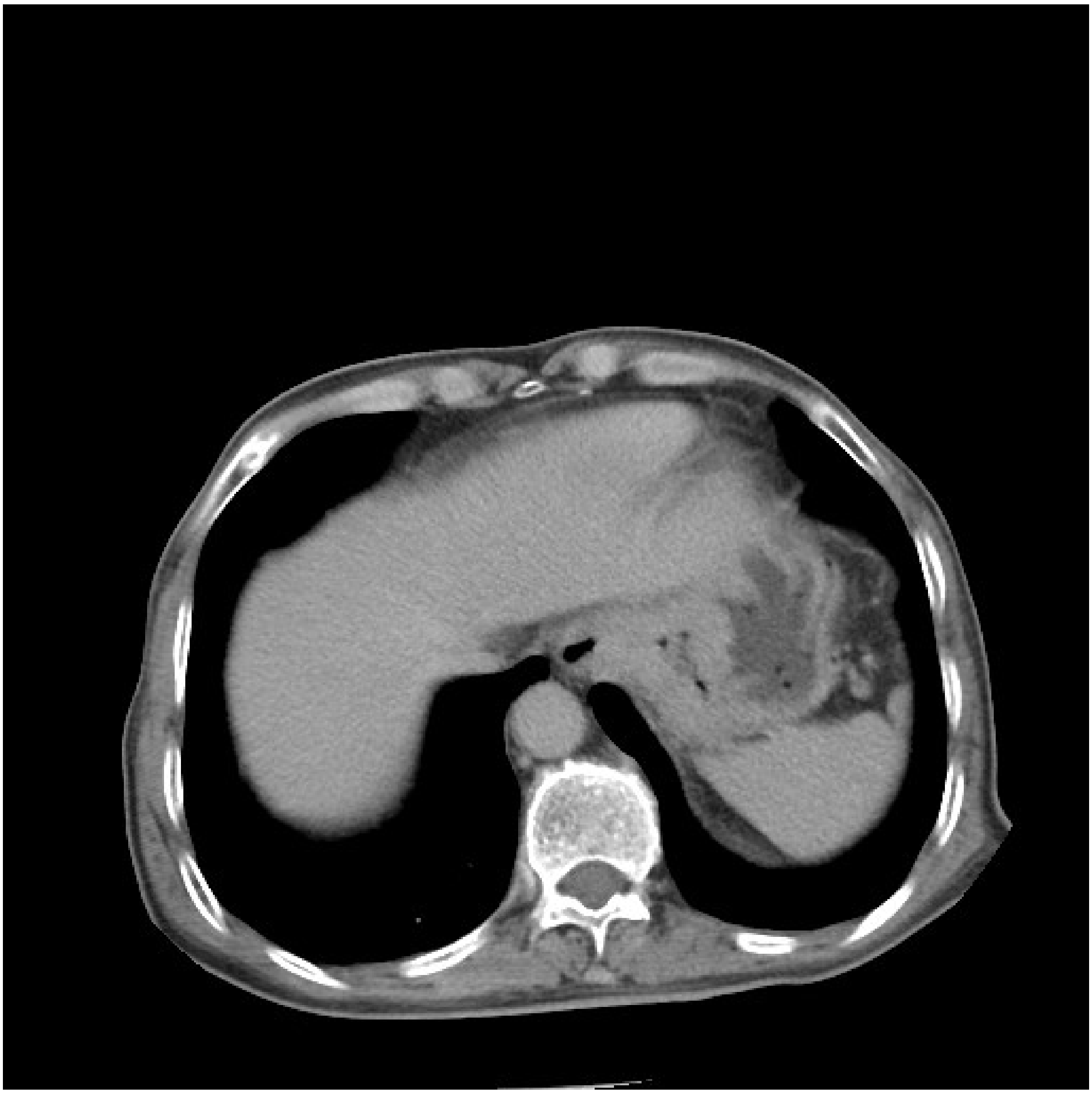

(**t**)

Figure S1. (**a**–**t**) Examples of patients with gastric cancer.

Here are a few examples showcasing the segmented tumor regions on a single CT slice.

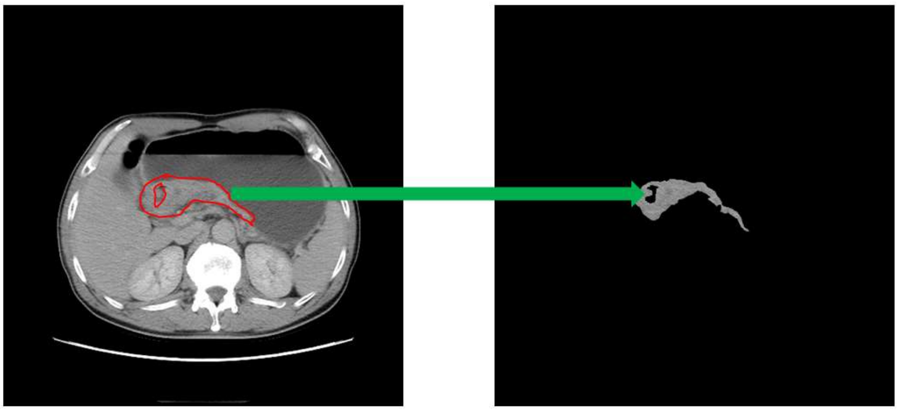

(**a**)

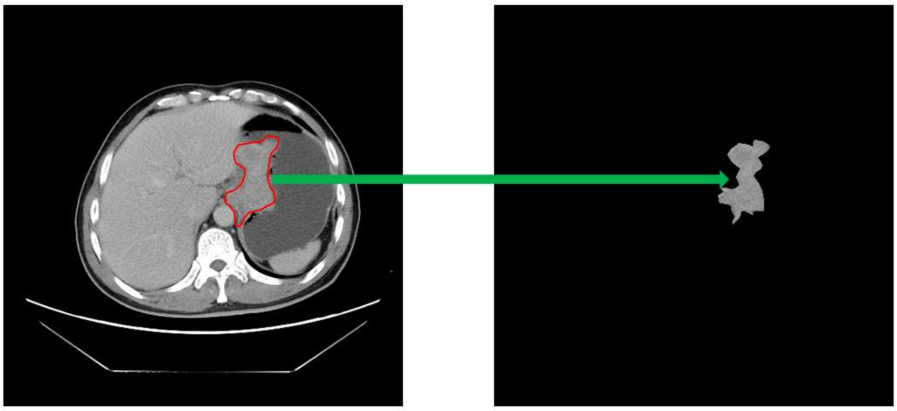

(**b**)

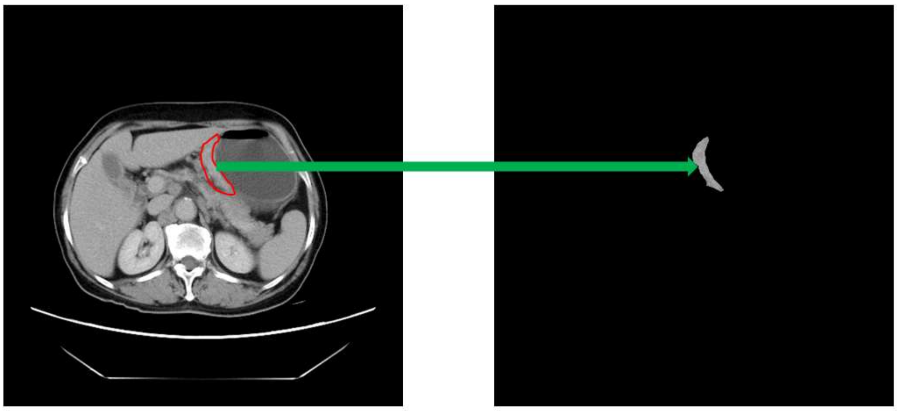

(**c**)

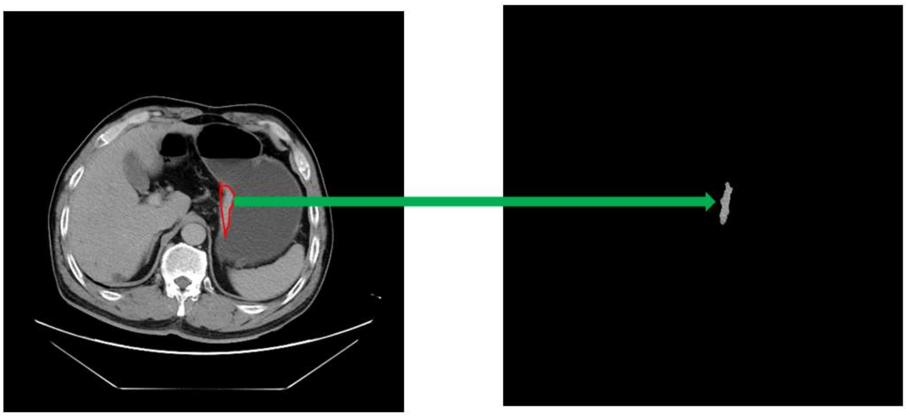

(**d**)

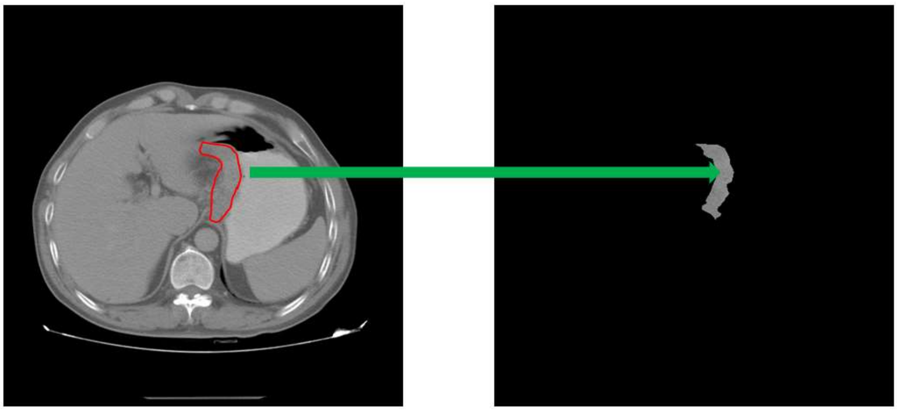

(**e**)

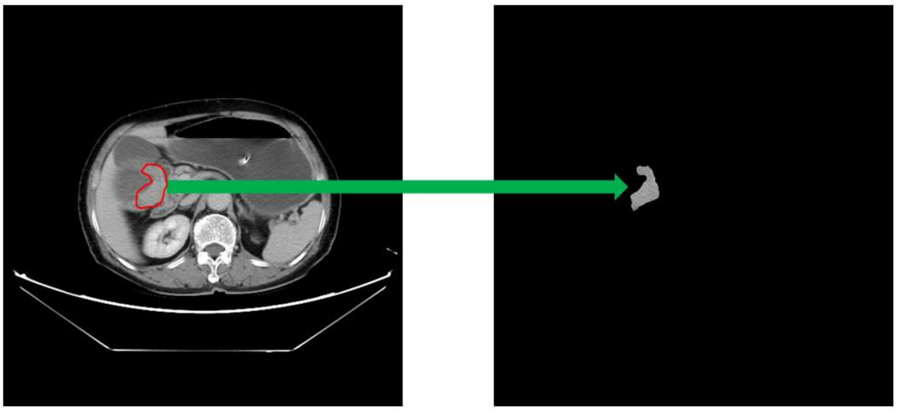

(**f**)

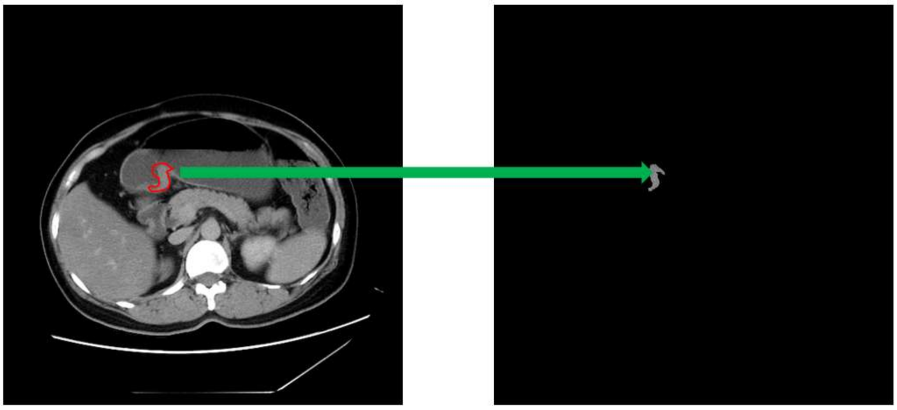

(**g**)

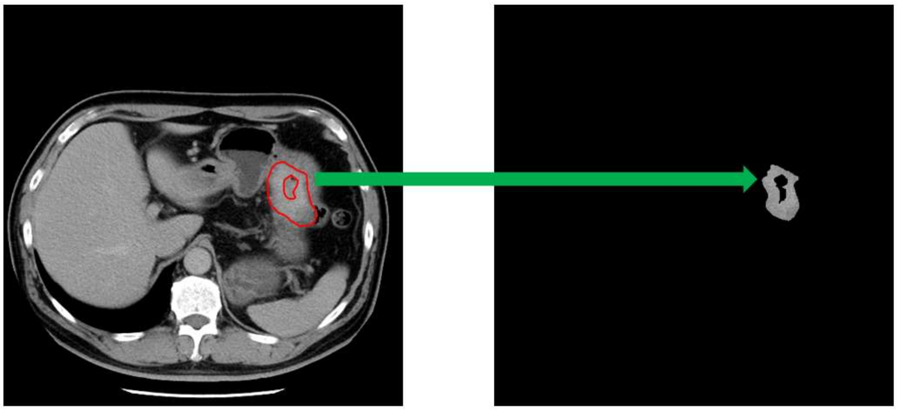

(**h**)

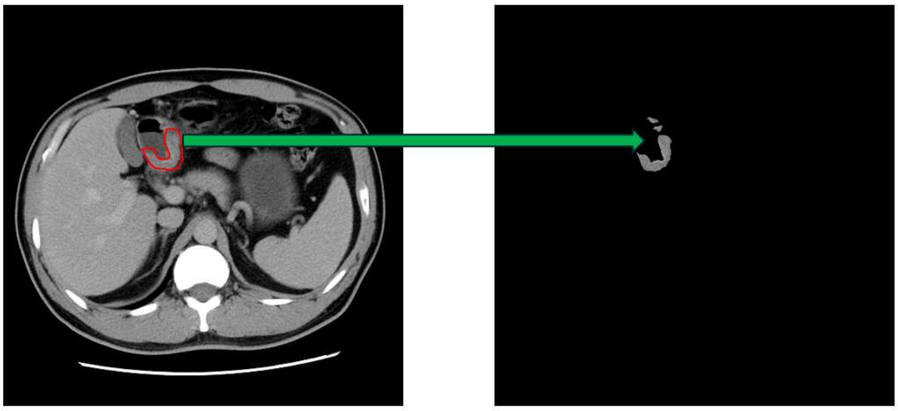

(**i**)

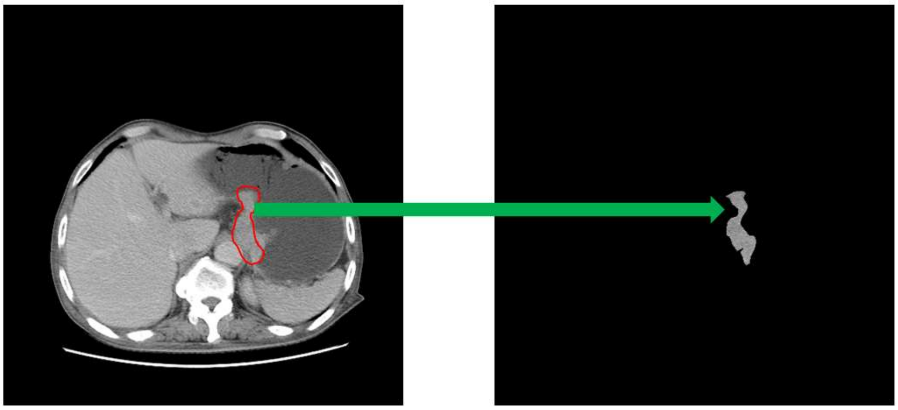

(j)

Figure S2. (**a**–**j**) Examples of illustrating the segmented tumor regions on one CT slice.
